# Millipedes as Food for Humans: Their Nutritional and Possible Antimalarial Value—A First Report

**DOI:** 10.1155/2014/651768

**Published:** 2014-02-12

**Authors:** Henrik Enghoff, Nicola Manno, Sévérin Tchibozo, Manuela List, Bettina Schwarzinger, Wolfgang Schoefberger, Clemens Schwarzinger, Maurizio G. Paoletti

**Affiliations:** ^1^Natural History Museum of Denmark, University of Copenhagen, Universitetsparken 15, 2100 Copenhagen Ø, Denmark; ^2^Dipartimento di Biologia, Università di Padova, lab. Agroecology and Ethnobiology, Via U. Bassi, 58/b, 35121 Padova, Italy; ^3^Escuela de Postgrado en Ciencias Biológicas, Universidad Nacional de Trujillo, Peru; ^4^Centre de Recherche pour la Gestion de la Biodiversité, 04 BP 0385 Cotonou, Benin; ^5^Institute for Chemical Technology of Organic Materials, Johannes Kepler University Linz, Altenberger Strasse 69, 4040 Linz, Austria; ^6^Institute for Inorganic Chemistry, Johannes Kepler University Linz, Altenberger Strasse 69, 4040 Linz, Austria

## Abstract

The first record of millipedes (Diplopoda) being regularly used for food by humans (the Bobo people of Burkina Faso) is given, including information on how the millipedes are prepared. The species in question are *Tymbodesmus falcatus* (Karsch, 1881) and *Sphenodesmus sheribongensis* (Schiøtz, 1966) (Gomphodesmidae) and an unidentified species of Spirostreptidae. New information on the nutritional value of millipedes is provided; unsaturated fatty acids, calcium, and iron contents are particularly high. The millipedes' defensive secretions, hydrogen cyanide and benzoquinones, present a severe challenge for the spread of millipedes as an everyday food source. On the other hand, the possibility that benzoquinones may act as insect-repellents, as known from studies on nonhuman primates, and that sublethal cyanide ingestion may enhance human innate resistance to malaria, suggests promising ethnomedical perspectives to our findings.

## 1. Introduction

Small vertebrates and invertebrates, especially insects, the so called minilivestock, have been considered a promising resource for Earth's human population that will reach 9 billion humans in 2050 [1–3] and are potential candidates for reducing the higher and increasing impact on resources represented by larger livestock and inland fish production [4]. Information on traditional local use of these small animals is an important starting point for studying minilivestock as potential food resources for humans [5–8]. In addition, local use of invertebrates may have unexpected ethnomedical implications.

Millipedes (Diplopoda) have so far not been in focus as minilivestock. Indeed, most orders of millipedes (Glomerida, Polyzoniida, Siphonocryptida, Platydesmida, Siphonophorida, Callipodida, Julida, Spirobolida, Spirostreptida, and Polydesmida) are known for their chemical defenses and, unlike their relatives, the centipedes (Chilopoda), which in several cultures (China, Alto Orinoco in Venezuela, and Korea) have been used as medical remedies and/or food items [9, 10], no information on millipedes as human food has been available until now. A wide spectrum of chemicals has been identified from millipede defensive secretions [11], the most widespread ones being benzoquinones (in most cylindrical millipedes, superorder Juliformia) and hydrogen cyanide derived from mandelonitrile and related compounds (in most flatbacked millipedes, superorder Merocheta). These toxic, smelly chemicals make millipedes unattractive for most predators, although there are some animals, vertebrates as well as invertebrates, which eat millipedes [12, 13], and some are even specialized on a millipede diet, for example, assassin bugs (family Reduviidae) of the subfamily Ectrichodiinae [14] and beetle larvae of the family Phengodidae [15]. A few vertebrates are reported to eat millipedes as for instance banded mongoose (*Mungos mungo*) [16]. Some birds and nonhominid primates use toxic millipedes for “self-anointment,” presumably exploiting an insect-repellent effect of the millipedes' defensive chemicals, especially benzoquinones [17–20]. We have, however, not been able to trace any record of millipedes being used as food in any human society, but now we can report the consumption of millipedes by the Bobo population of Burkina Faso, a region where entomophagy has been extensively described [21].

The millipedes which are used as human food by the Bobo belong to two families: Gomphodesmidae and Spirostreptidae.

**Figure 1:**
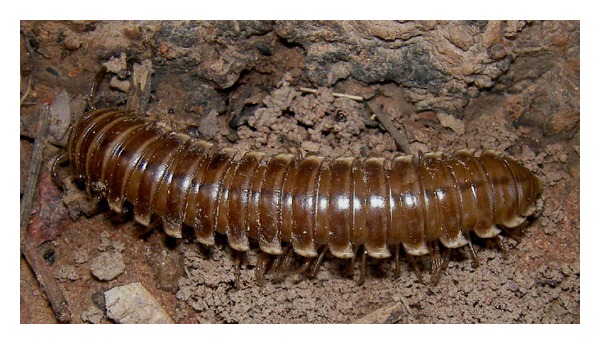
Live gomphodesmid millipede (*Tymbodesmus falcatus*) from Kou. S. Tchibozo phot., 2011.

**Figure 2:**
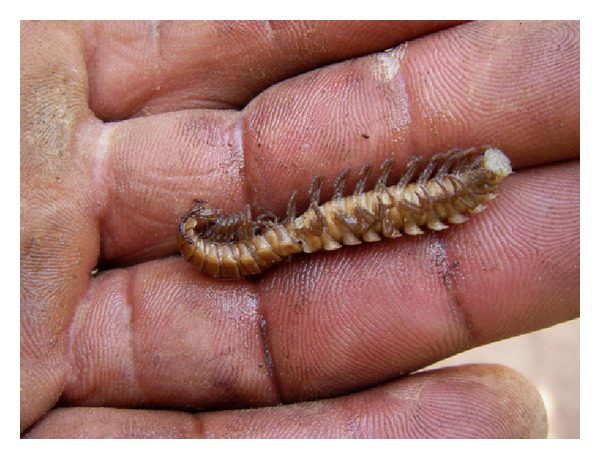
gomphodesmid millipede from Kou after boiling. S. Tchibozo phot., 2012.

Gomphodesmidae (flatbacked millipedes of the order Polydesmida) (Figures 1 and 2) belong to the “cyanogenic” millipedes; the family is endemic to the African continent south of the Sahara, includes 146 named species [22], and was monographed by Hoffman (2005) [23]. The only gomphodesmid species previously recorded from Burkina Faso is *Tymbodesmus falcatus *(Karsch, 1881), collected in Ouagadougou [23]. One of the gomphodesmid species used for food at Kou village in Burkina Faso is indeed *T. falcatus *(D. Vandenspiegel det.), the other is *Sphenodesmus sheribongensis *(Schiøtz, 1966) (HE det.). *T. falcatus *is known from Mali, Burkina Faso, Nigeria, Sudan, and Central African Republic; *S. sheribongensis *was previously known from Ghana, Ivory Coast, and Nigeria [23]. Lewis [24] studied the life history and ecology of both species in Zaria, Northern Nigeria. Both have a two-year life cycle. Juvenile stadia of *S. sheribongensis *live entirely in the soil, whereas adults can be extremely abundant on the soil surface during the first part of the rainy season (from May to July). *T. falcatus *is similar, except that the last juvenile (subadult) stadium is seasonally surface active like the adults, albeit in smaller numbers.

**Figure 3:**
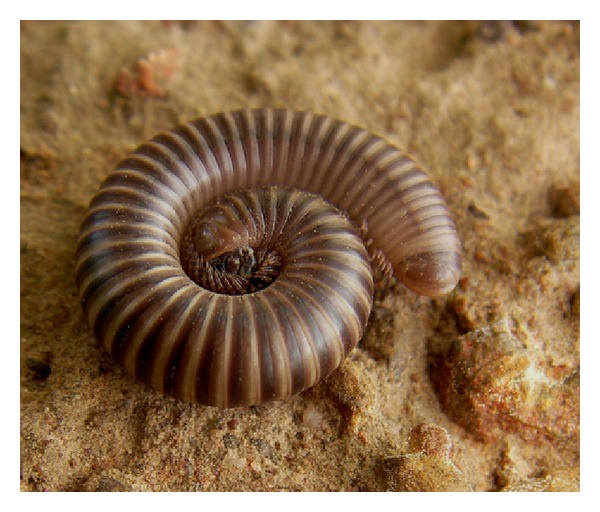
Spirostreptid millipede from Kou. S. Tchibozo phot., 2012.

Spirostreptidae (cylindrical millipedes of the order Spirostreptida) (Figure 3) belong to the “quinone” millipedes: the family is near-endemic to the Afrotropical and Neotropical regions, includes 275 named species [22], and was monographed by Krabbe [25]. No species of Spirostreptidae have been recorded from Burkina Faso, and the one occurring at Kou has not yet been identified.

We do not have any strong evidence that *T. falcatus* and *S. sheribongensis* actually produce hydrogen cyanide, nor that the spirostreptid in question produces quinones. The general occurrence of these substances in the respective higher taxa to which the species belong, however, is strong circumstantial evidence that this is actually so, although recent studies have demonstrated a larger diversity in millipede defensive chemicals than previously assumed [26–28].

Until this study, the nutritional value of millipedes has never been assessed. Considering that the muscle volume of millipede is small, they will constitute a poor source of protein. Their guts mainly contain soil and litter remains [24], but their calcified exoskeleton might constitute a considerable source of calcium, which may constitute 13–17% of the dry weight [29–31] or 9% of the fresh weight [32], In fact, millipedes are considered an essential source of calcium for egg production in certain birds [30, 33, 34]. In this work we present data based on *Tymbodesmus falcatus, *one of the species eaten by the Bobo people.

## 2. Material and Methods 

### 2.1. Sampling and Ethnobiological Data Collection

Observations and interviews with the Bobo people were made by ST in 2011 and 2012 in Burkina Faso (Kou, 11°10.88′ N, 004°26.62′ W, altitude 351 m, near Bobo-Dioulasso). Exemplars of the edible millipedes were collected and are now preserved in Musée Royale de l'Afrique Centrale (Tervuren, Belgium) and the Natural History Museum of Denmark (Copenhagen). Five Bobo people, especially women, were interviewed for information on collection and preparation of the edible millipedes.

### 2.2. Nutritional Analysis

In order to assess the nutritional value of this unconventional food we determined various nutritional parameters of a whole *Tymbodesmus falcatus *male specimen. The raw specimen was preserved in 70% ethanol, subsequently dried and homogenized by cryomilling (SPEX Freezer/Mill 6770). The resulting powder was used for analysis of chitin, fatty acids, amino acids, and metal content. No appropriately preserved specimens of the other species in question were available. Dry weight of millipedes was measured on oven-dried specimens and kept in alcohol for 45 years (collected in Nigeria by J.G.E. Lewis).

#### 2.2.1. Pyrolysis-GC/MS for Fatty Acid Analysis

100 *μ*g of the sample was placed in a quartz tube, and 4.5 *μ*L of a diluted, aqueous solution of tetramethylammonium hydroxide was added. The samples were subsequently pyrolyzed at 450°C for 10 s with a CDS 5250 pyrolysis autosampler attached to a Thermo Trace GC Ultra/MD 800 gas chromatography/mass spectrometry system. Volatile products were separated on a Supelco SP 2330 column (30 m, ID 0.32 mm, 0.2 *μ*m film thickness) with helium 4.6 as carrier gas (2 mL·min^−1^) and identified by comparison to reference compounds as well as interpretation of their EI mass spectra and comparison to NIST 2002, Wiley, and NBS electronic libraries. The pyrolysis interface was kept at 300°C and the GC/MS interface at 280°C; the GC was programmed from 100°C (1 min) to 230°C (5 min) at a rate of 10°C min^−1^. The mass spectrometer was operated in EI mode (70 eV) at a source temperature of 200°C [35].

#### 2.2.2. Solid State NMR for Chitin

Chitin was determined by Solid State NMR; all spectra were recorded on a narrow-bore 11.7 T instrument (500 MHz, 1 H Larmor frequency) at magic angle spinning rates of 10.0 kHz at 300 K. 13C chemical shifts are given in reference to tetramethylsilane TMS, using the sharp resonance of TMS as external calibration. A basic cross polarization experiment with total suppression of sidebands with a cross-polarization contact time of 2 ms was employed, with an effective acquisition time of 27.9 ms and a recycling delay of 5 s. The magic angle was adjusted using the 79Br resonance of KBr, and the actual sample temperature was determined using the ^207^Pb resonance of Pb(NO_3_)_2_ for calibration [36].

#### 2.2.3. Amino Acid Analysis

The powder obtained after cryomilling was hydrolysed by refluxing with hydrochloric acid containing 5% phenol for 24 hours under exclusion of oxygen. The complete sample was then evaporated to dryness, redissolved in water, and analyzed with HPLC/MS using 0.5 mL·min^−1^ of a water acetonitrile gradient (100% water for 2 minutes and in 17 minutes to 30% acetonitrile which is held for a further 3 minutes) on a Waters AccQ Tag column (3.9 × 150 mm). Quantification was done using extracted ion chromatograms. With this procedure arginine, cysteine, and histidine could not be analyzed and leucine/isoleucine as well as glutamine/lysine could not be separated; therefore, the concentration of those amino acids is given as a sum.

#### 2.2.4. Metal Analysis

Metal content was analysed with inductively coupled plasma optical emission spectrometry (ICP-OES) according to EN ISO 11885 from a commercial laboratory.

## 3. Results 

### 3.1. Collection and Preparation of Millipedes at Kou Village

According to the interviewed villagers in Kou, millipedes are collected under bricks around houses made of straw and under decomposing wood. Once collected, the millipedes are placed in a pot with water filtered through firewood ashes, for 3–5 minutes until boiling. Then they are removed and left to dry on a roof for 3 days. Such preparation is specific for millipedes and different from those described for other arthropods in West Africa, especially for insect maggots and weevils, which are mainly roasted and fried [21]. The dried millipedes are placed in a tomato sauce to which is added the traditional African mustard known as *soumbala *(fermented seeds of the *néré *tree, *Parkia biglobosa*, very widely consumed in Burkina Faso and West Africa in general), *shea *butter oil, and *tô *(a paste made from maize or sorghum flour). For some meals, the millipedes replace meat.

### 3.2. Nutritional Values of *Tymbodesmus falcatus*

**Figure 4:**
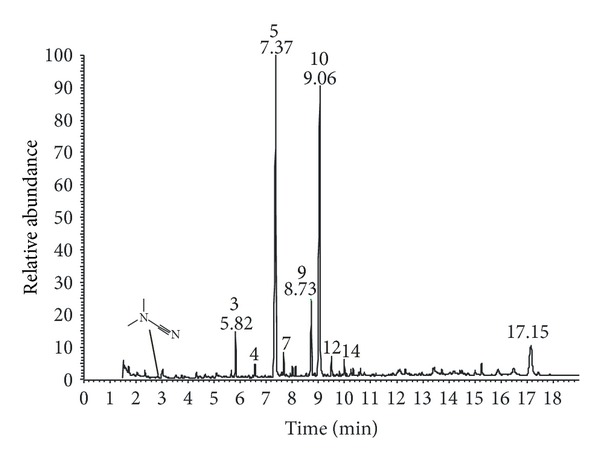
THM-GC/MS profile of *Tymbodesmus falcatus*; retention times (*t*_*R*_) refer to compounds listed in Table 2: palmitic acid 18 : 1 n9 (*t*_*R*_: 7.37) (5) and oleic acid 16 : 0 (*t*_*R*_: 9.06) (10) constitute 80% of total fatty acids.

Proteins represent 25% of total dry weight (calculated as the sum of amino acids); the amino acid profile (Table 1) is similar to that of insects and crustaceans, for example, crickets and shrimps [37]. Unsaturated fatty acids constitute a relevant fraction (40%) of total fatty acids (Table 2, Figure 4), however lower than those described for widely consumed and appreciated edible insects [38]. Calcium levels (Table 3) are very high (17.4% of dry weight) which is higher than previously published values [29–32].

The dry weight of individual *Tymbodesmus falcatus* was 0.42–0.54 g (mean 0.46, *n* = 3) and of the smaller *Sphenodesmus sheribongensis,* 0.08–0.11 g (mean 0.09, *n* = 4). Spirostreptidae vary very much in size; live weights of up to 80 g have been measured (HE unpublished). Considering that the Ca content of a single *Tymbosdesmus f.* is about 80–90 mg (174 mg/g × 0.46 g), consumption of around 12-13 gomphodesmid individuals will provide 1000 mg/day, (the Dietary Reference Intake (DRI), by IOM 2004 [39]). Also iron content (100,600 mg/kg) is important considering that only 6 individuals provide the adequate amount for a women during pregnancy (DRI: 27 mg/day) and only 2-3 for men (DRI: 8 mg/day) [39].

Chitin constitutes around 5% of the total dry weight, a low amount compared to Ca, but it is only located in the exoskeleton (incl. legs).

A trace level of dimethylcyanamide revealed in the GC/MS (see Figure 4) is the only direct evidence of cyanogenic compounds in our sample.

## 4. Discussion 

The gomphodesmid millipedes utilized by the Bobo people are quite small (max. 5 cm long), but gomphodesmids may occur in very large numbers at certain times of the year. Thus, Lewis [24] stated that 50 specimens of *S. sheribongensis *could be collected in 10–15 minutes at the beginning of the rainy season in Zaria, Nigeria and even (pers. comm.) that it was sometimes impossible to take a step without crushing several individuals. Barbetta et al. [40] used 1200 specimens of *Haplogomphodesmus pavani *(Demange, 1965) for their biochemical study, an indication that also this species can be quite abundant. Spirostreptids may occasionally form huge swarms, for example, in Ghana and Zimbabwe [41]. They are sometimes of considerable size, up to 30 cm long. Considering the regular and high levels of some essential nutrients (Ca, Fe, PUFA) and seasonal abundance of millipedes, they likely represent a significant type of minilivestock for the Bobo people. The ethnobiological/ethnopharmacological uses of many unconventional species, that is, earthworms and insects, are documented in several cultures, being part of a complex system of specific traditional knowledge adapted to local resource availability and to very variable hygienic standards [42, 43] that may not fit with the food-quality/food-safety standards imposed in industrialized countries [44].

As mentioned above, gomphodesmids belong to the “cyanogenic” millipedes [11]. Cyanogenesis in millipedes and other arthropods has not been studied with state-of-art methodology [41], but the presence of hydrogene cyanide and its precursors has been demonstrated in many species of the order Polydesmida [11, 26, 27, 45, 46], including two species of Gomphodesmidae; HCN has been identified in the secretion of *Astrodesmus laxus *(Gerstäcker 1873) from East Africa [47], and its precursor, mandelonitrile, in *Haplogomphodesmus pavani *[40]. Although there is no direct information on the secretions of *T. falcatus *and *S. sheribongensis*, there is no reason to believe that they have lost the cyanogenic function. They have the same complement of defense glands as the majority of gomphodesmids, including *A. laxus *and *H. pavanii,* that is, 11 pairs of glands opening along the sides of the body [23].

Benzoquinones have been detected in several spirostreptid species [11, 28, 48]. In Figure 3 the defensive glands can be seen as a series of darker spots, one on each diplosegment except the very first and last ones.

Some birds and mammals (see Table 4) have been described to use “quinone” millipedes for self-anointing in order to control ectoparasites and mosquito biting rate, and some mammals like opossum, coatis, skunk, mongoose, and lemurs are known to consume toxic millipedes—mainly Spirostreptidae—after different treatments such as prey-rolling, handling, and salivating [13, 49–52]. The complex behaviours that precede ingestion require a considerable investment of time and energy and are evidently necessary, maybe in order to reduce toxicity of the millipedes to be eaten [53, 54]. The use of benzoquinonigenic plants for self-anointing in orangutans [54] and owl monkeys [55] supports the hypothesis that benzoquinones are used by primates for their specific biochemical defensive properties. Contrarily, use and consumption of gomphodesmid cyanogenic millipedes seems to be rare even in most omnivorous mammals, except opossum [56].

### 4.1. Cyanide in Traditional Foods

The use of millipedes as human food is absolutely exceptional, but the use of food items containing significant levels of cyanide is widespread [26]. Over 2000 plant species contain cyanide as a defense against insects and other herbivores [26] and the most important cyanogenic crop is cassava (*Manihot esculenta*, Crantz), a staple food of hundreds of millions of humans in the tropics [57, 58].

In the Amazons—the centre of domestication of cassava—the more toxic bitter varieties named *yuca amarga* are the most intensively cultivated because of their resistance to pest insects and rodents. However, cassava domestication largely preceded malaria ingression in the New World [59] and the indigenous preparation is aimed at avoiding the cyanogenic compounds, consisting of specific postharvest operations: grinding, squeezing, toasting, and fermentation [58, 60]. Boiling alone is not enough to avoid the toxicity of *yuca amarga *[58], and only the sweet varieties of *yuca dulce*, which are low in glycosides, can be consumed safely in soups or fried (MGP personal observation in Alto Orinoco, Venezuela).

The Bobo people subboil the millipedes, as part of the preparation for meals. This treatment may degrade the cyanogenic compounds by releasing the hydrogen cyanide gas, thereby detoxifying the gomphodesmids.

Nonetheless, the short (3–5 minutes) subboiling and natural drying appear to be a specific treatment for millipedes, maybe aimed at preserving part of their chemicals. Moreover, benzoquinones are not readily soluble in water and the most characteristic juliform benzoquinone, toluquinone, is insoluble in water [61]; thus substantial amounts of them may remain in the millipede bodies even after cooking.

### 4.2. Biocultural Perspective

African populations consume raw and subboiled bitter cassava [58, 60, 62, 63], as well as many other bitter food items that would not be tolerated by other populations. It has been proposed that the reduced sensitivity to bitterness is an ancient adaptation (70 kY BP) [64] of the human bitter-taste specific receptor to bitter antimalarial compounds (e.g., flavonoids), which are still abundant in the West African diet [62, 65]. Cassava contains the cyanogenic glucoside compounds linamarin and lotaustralin that, once ingested, are metabolized to thiocyanate and cyanate. Notably, these metabolites are biologically active although less toxic than cyanide and, at levels of expected dietary intake, cyanide-related compounds (e.g., cyanate) are able to modify essential proteins of *Plasmodium falciparum* and inhibit parasite survival [66].

Considering that malaria represents the main cause of mortality in the adult sub-Saharan population and that Burkina Faso is endemic for several *Plasmodium *species [67, 68], biocultural adaptations aimed at controlling this pathogen are strongly expected. Natural benzoquinones are known to exert an antiplasmodic activity in vitro [69], and their metabolites might act as systemic repellents against mosquitoes or as antimalarial prophylaxis in the Bobos. Moreover, studies on West African populations have demonstrated strong links between malaria, sub-lethal cyanide intake from bitter cassava, and sickle-cell anemia [60, 70], a genetic pathology affecting erythrocytes that confers protection against *Plasmodium*. Thus, after demonstrating that cyanide interacts with hemoglobins, partially compensating sickle-cell dysfunctionality [60, 71], some authors proposed that the abundant consumption of bitter foods could enhance biological fitness in West African populations exposed to malaria [62, 70]. Therefore, both ethological and bioanthropological evidences suggest that “toxic” millipedes consumed by the Bobo people take part in a complex biocultural mechanism for malaria control.

## 5. Concluding Remarks

**Figure 5:**
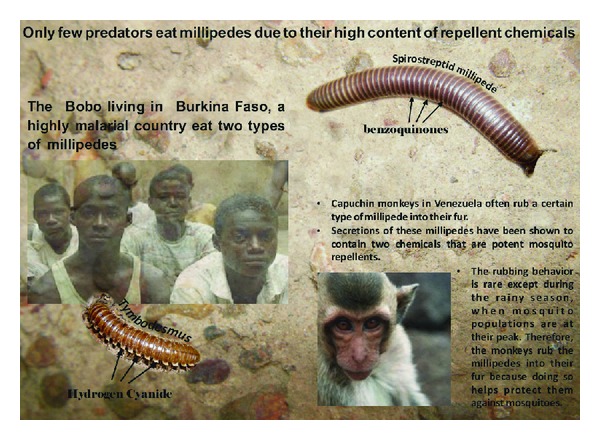
Scheme of the exceptional use of toxic millipedes: as demonstrated for capuchin monkeys, that during the rainy season use millipedes' secretions against mosquitoes, Bobo people in Burkina Faso may consume millipedes for their antiparasite effect.

Key topics of this paper are summarized in Figure 5. Whether millipedes will ever become a major actor in minilivestock husbandry may be dubious, but the Bobo people have shown that they constitute a helpful food source for an ever-growing human population, especially in rural Africa. In addition, the potential of millipede chemicals for deterring mosquitoes and for influencing *Plasmodium *and other parasites constitutes a promising field of research.

## Figures and Tables

**Table 1:** *Tymbodesmus falcatus* amino acid analysis (based on dry matter).

Amino acid	mg/mg
Alanin	0.0676
Asparagin	0.0137
Asparaginic acid	0.0164
Glutamin + lysin	0.0217
Glutamic acid	0.0000
Glycin	0.0000
Isoleucin + leucin	0.0283
Methionin	0.0056
Phenylalanin	0.0132
Prolin	0.0062
Serin	0.0142
Threonin	0.0251
Tryptophan	0.0053
Tyrosin	0.0151
Valin	0.0193

Total	0.2518

**Table 2:** Fatty acid distribution as determined by pyrolysis-GC/MS analysis. Unsaturated fatty acids constitute 40% of total fatty acids of *Tymbodesmus falcatus*.

No.	ID	Name	*t*_*R*_	Percent
1	10:0	Caprylic acid	3	0.3
2	12:0	Lauric acid	4.33	0.5
3	14:0	Myristic acid	5.82	3.1
4	15:0	Pentadecanoic acid	6.56	1.0
5	16:0	Palmitic acid	7.37	43.1
6	16:1 n9	Sapienic acid	7.61	0.2
7	16:1 n7	Palmitoleic acid	7.67	1.5
8	17:0	Margaric acid	8.01	0.8
9	18:0	Stearic acid	8.73	7.8
10	18:1 n9	Oleic acid	9.06	36.8
11	18:1 n7	Vaccenic acid		0.0
12	18:2 n6	Linoleic acid	9.5	1.6
13	18:3 n6	Gamma linolenic acid		0.0
14	20:0	Arachidic acid	10	1.1
15	18:3 n3	Alpha linolenic acid	10.08	0.1
16	18:2 x	Octadecenoic acid	10.24	0.5
17	20:1 n9	Gadoleic acid		0.0
18	18:2 x	Octadecenoic acid	10.34	0.5
19	18:2 x	Octadecenoic acid	10.55	0.3
20	20:2	Eicosadienoic acid		0.0
21	20:3	Eicosatrienoic acid		0.0
22	22:0	Behenic acid	11.2	0.1
23	20:4 n6	Arachidonic acid		0.0
24	21:0	Heneicosylic acid	11.77	0.1
25	20:5 n3	Timnodonic acid		0.0
26	24:0	Lignoceric acid	12.33	0.4
27	22:5 n6	Docosapentaenoic acid		0.0
28	22:6 n3	Docosahexaenoic acid		0.0

Total				100.0

**Table 3:** *Tymbodesmus falcatus* metal contents (based on dry matter) and DRIs for a pregnant women of 19–30 yrs, by IOM 2004 [39].

Metal	mg/kg	DRI (mg/day)
Pb	6.2	—
Cd	<0.5	—
Ca	174,000	1,000
Fe	10,600	27
K	2,610	4,700
Cu	789	1
Mg	4,990	350
Na	1,630	1,500
Zn	160	11

**Table 4:** Summary of the current knowledge about utilization of millipedes by mammals as anointing and/or food item.

Mammals	Millipedes	Chemicals	Use	Reference
Marsupialia				
Opossum (*Didelphis albiventris*)	*Leptodesmus dentellus *(Chelodesmidae) *Gymnostreptus olivaceus* (Spirostreptidae)	Benzoquinones and Cyanogenics	Consumption and sniffing	[47]

Carnivora				
White-nosed coatis (*Nasua narica*)	*Orthoporus* sp. (Spirostreptidae)	Benzoquinones	Consumption after prey-rolling treatment	[13]
Meerkat-mangoose (*Suricata suricatta*)	?	?	Consumption after treatment	[49, 51]
Striped skunk (*Mephitis mephitis*)	?	?	Consumption	[52]

Primates				
Capuchin monkeys (*Cebus* sp.)	*Orthoporus dorsovittatus* (Spirostreptidae)	Benzoquinones	Self-anointing	[19]
Capuchin monkeys (*C. olivaceus*)	*Orthoporus dorsovittatus* (Spirostreptidae)	Benzoquinones	Self-anointing	[18]
Owl monkeys (*Aotus* sp.)	*Anadenobolus monilicornis* (Rhinocricidae)	Benzoquinones	Self-anointing	[55]
Black lemurs (*Elemur macaco*)	*Charactopygus* sp. (Spirostreptidae)	Benzoquinones ?	Self-anointing	[17]
Lemurs (*Varecia rubra* and *Eulemur fulvus albifrons*)	?	?	Self-anointing and consumption after handling and salivating	[50, 53, 72]
Humans, Bobo population	*Tymbodesmus falcatus *and* Sphenodesmus sheribongensis* (Gomphodesmidae) unknown Spirostreptidae	Benzoquinones and Cyanogenics	Consumption after boiling and drying	This study
